# Beware of the ambiguous enemy of multisystem inflammatory syndrome in adult (MIS‐A) following Covid‐19 infection or vaccination

**DOI:** 10.1002/ccr3.5138

**Published:** 2021-11-26

**Authors:** Ahmad Al Bishawi, Maisa Ali, Khaled Al‐Zubaidi, Hamad Abdelhadi

**Affiliations:** ^1^ Infectious Diseases Division Department of Internal Medicine Communicable Diseases Centre Hamad Medical Corporation Doha Qatar; ^2^ Department of Paediatrics Paediatric Infectious Disease Hamad Medical Corporation Doha Qatar

**Keywords:** COVID‐19, cytokine storm, MIS‐A, SARS‐CoV‐2, sepsis

## Abstract

Multisystem Inflammatory Syndrome is a rare and novel clinical presentation described during the evolving COVID‐19 pandemic. The condition is usually presenting as a sepsis‐like syndrome leading to secondary multi‐organ dysfunction post–COVID‐19 infection. Although the syndrome has been mainly described in children, rare adults' form has been similarly described. We are describing a 37‐year‐old female patient presented with fever and neck pain after 1 month of a mild SARS‐CoV‐2 infection course and 10 days post her second COVID‐19 vaccine. Examination demonstrated fever, hypotension, and hypoxemia, in addition to multiple tender cervical lymph nodes. Initial laboratory workup showed evidence of significant inflammation with raised markers, including C‐reactive protein, ferritin, and interleukin‐6. Extensive evaluation to rule out active infection was done, and all return negative, including repeat SARS‐CoV‐2 test. Furthermore, cardiac evaluation showed moderately reduced systolic ventricular function. Despite all negative test and supportive measures, the patient continued to deteriorate requiring critical care admission for ionotropic support, non‐invasive ventilation in addition to presumptive broad‐spectrum antimicrobial management. There was no significant improvement with supportive care until the presentation of multisystem involvement on in the context of a recent history of COVID 19 and negative infective screen was raised. The diagnosis of multisystem inflammatory syndrome‐adult form (MIS‐A) was embraced, and the patient was commenced on methylprednisolone leading to a dramatic resolution of symptoms both clinically and biochemically with stabilization of vital functions allowing for safe outcomes.

## Graphical abstract

MIS‐A should be considered with a sepsis‐like syndrome following a recent COVID‐19 disease or vaccination and there are no alternative diagnoses. Steroid might be beneficial in suppressing the hyper‐immune activation in such population.

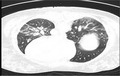

## 1 BACKGROUND

COVID‐19 disease originated in China in late 2019 and has been declared as a pandemic by the WHO in March 2020. The pneumotropic virus led to a global pandemic with significant morbidity and mortality mainly secondary to respiratory complications including predominant parenchymal lung disease with secondary respiratory complications including pneumonia, acute respiratory distress syndrome (ARDS) and occasional intense immune activation associated with multi‐organ failure.^1^ Despite the role of the secondary immune activation leading to multiple organs involvement was recognized early during the pandemic, the complete role of the immune system in post COVID‐19 infection or vaccinations sequalae has not been fully evaluated. Consequently following established viral infection, clinical presentation with hyper‐immune dysfunction rose to the surface with sepsis‐like syndrome and multi‐organ dysfunction mainly in children and young adolescents described as multi‐system inflammatory syndrome in children (MIS‐C).^2^ Fortunately, although the condition carries significant morbidity frequent in need of critical care supportive therapy, mortality remains low.^3^ The condition has a set criterion for evaluation that mainly entails exclusion of other potential causes such as persistent viral infections including COVID‐19 disease or secondary bacterial complications as well as fulfillment of diagnostic criteria.^4^, ^5^

While the majority of causes were described in the pediatric population, some cases have been reported in adults leading to the mirrored nomenclature of MIS‐A.^6^ Since the condition is rare but ominous, clinical awareness is needed to allow of early recognition to expedite evaluation toward appropriate management. In this case report, we outline the case of an adult with a recent SARS‐CoV‐2 infection and COVID‐19 vaccination who presented with stormy presentation mimicking severe sepsis which was managed accordingly but with multiple negative tests toward alternative diagnoses till MIS‐A was eventually considered leading to a safe outcome. The role of management including steroid suppressive therapy and other immune modulating agents will be evaluated against available evidence.

## 2 CASE PRESENTATION

Our patient is a 37‐year‐old Jordanian female patient who was previously well with no significant past medical history. Following the evolving pandemic, in April 2021 she received the first dose of Modern mRNA vaccine; however, 2 weeks afterward she was exposed to a COVID‐19 positive case and started complaining of fever and mild non‐productive cough. She tested positive for SARS‐CoV‐2 with three targets‐based RT‐PCR (S, N, and E) with a cycle‐ threshold (CT) value of 21.7 denoting recent acquisition. Evaluation with baseline laboratory tests including complete blood and biochemical tests as well as inflammatory markers in addition to a chest radiography were within normal limits. As per local protocol, she was confined to home isolation for 14 days as a non‐complicated infection. Following completing the planned fortnight quarantine uneventfully, she went to receive the second dose of the vaccine.

On the 25^th^ day following the COVID‐19 infection and the 10^th^ following the second dose of vaccination, the patient presented to our acute care hospital with a chief complaint of documented fever of 40°C accompanied by chills as well as residual cough and significant neck pains. Upon evaluation, the patient mentioned mild dyspnea but denied any other focal systemic symptoms. Extended history revealed, no relevant recent travel history, being a teetotal with no other recreational habits, she also denied any animal or other significant infective exposures.

Upon initial evaluation, the patient was febrile with oral temperature of 39°C, pulse rate of 115 beat per minute, blood pressure of 97/58 mmHg, and a respiratory rate of 21 breath per minute with oxygen saturation of 95% on ambient air. Physical examination revealed alert oriented and conscious patient but with overall sick general appearances. There were no meningeal signs, and her ear, nose, and throat examinations were normal. Local neck examination revealed multiple tenders right sided cervical and axillary lymphadenopathy while her chest was clear to auscultation and heart sounds should only tachycardia with no added sounds. Abdomen was soft and lax with no tenderness.

Blood results showed bi‐cytopenia with hemoglobin of 9.4 gm/dL, platelet of 137 × 10^3^/μL, and while blood cells count was 5.8 × 10^3^/μL but with lymphopenia (Table 1). Other significant laboratory test showed D‐ Dimer of 1.99 mg/L, ALT of 50 U/L, raised inflammatory markers in form of C‐Reactive protein of 237 mg/L, procalcitonin of 0.81 ng/mL, and ferritin 471.0 μg/L. Additional negative test included normal rest of coagulation panel, renal functions, malaria film smears as well as cardiac troponin enzymes as well as HIV and Brucella serology. To investigate for potential respiratory infections, full respiratory panel in form of EBV/CMV/Adeno viruses PCR were all negative including a repeat COVID‐19 PCR test.

**TABLE 1:** Laboratory results upon initial evaluation and subsequent critical care admission

Results	Medical ward	Critical care	Normal references
WBC	5.8	8.8	4–10 × 10^3^/μL
HGB	9.4	8.3	12–15 gm/dL
PLT	137	167	150–400 × 10^3^/μL
Creatinine	46	36	44–80 μmol/L
Na	138	137	135–145 mmol/L
ALT	50	29	<40 U/L
TSB	19	26	<21 μmol/L
CRP	237	302	<5 mg/L
Procalcitonin	0.8	2.4	<0.05 ng/mL
Troponin	<3	463	<10 ng/L

**FIGURE 1:**
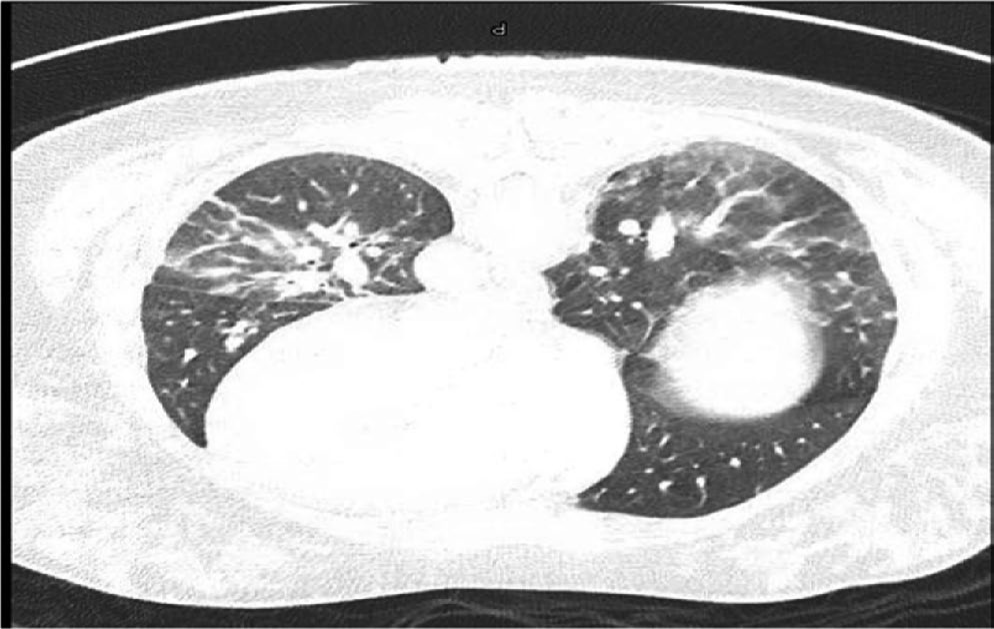
High‐resolution CT pulmonary angiogram showed no evidence of pulmonary embolism but bilateral ground glass opacities

In view of the recent COVID‐19 disease, hypotension, tachycardia, and raised D‐Dimer results, CT pulmonary angiogram was performed to exclude potential pulmonary embolism. The condition was excluded but revealed bilateral pulmonary ground glass and consolidative changes with accompanied splenomegaly attributed to the recent disease (Figure 1). Following admission to the medical wards, the patient continued to be febrile with hypotension and hypoxia requiring minimal oxygen supplementation. Septic work up in the form of multiple sets of blood, urine, and sputum cultures were obtained followed by empirical antimicrobial coverage with Piperacillin Tazobactam and Azithromycin in addition to intravenous fluid challenge. Despite these supportive measures, the patient's condition deteriorated and continued to be hypotensive necessitating admission to critical care where she was started on vasopressor support with the main evaluation as complicated sepsis syndrome while repeat clinical examination did not change significantly from admission evaluation. Repeat laboratory test (Table 1) showed progression of inflammation with CRP raised to 326.1 mg/L, procalcitonin to 1.67 ng/mL, ferritin of 576.0 μg/L, and Interlukin‐6 to 86 pg/mL. Screening for other causes of significant inflammations with ANA, C‐ANCA, C3, and C4 were within normal limits while cardiac markers signified acute injuries with serial rise of cardiac troponins up to of 947‐ng/L, and Pro BNP to 7479 pg/mL in the absence of electrocardiograms changes of acute pathology. Echocardiography demonstrated moderately reduced left ventricular function with EF of 40% and global hypokinesia evaluated as sepsis related myocarditis and managed accordingly.

While evaluation for sepsis was the main objective, it was not supported with confirmatory results with multiple negative tests for underlying infective processes including repeat molecular tests and non‐response to conventional management of sepsis. When the patient condition continued to deteriorate, the presentation of multisystem involvement with no alternative identifiable source in the context of a history of both COVID‐19 infection and vaccination, the possibility of MIS‐A was considered, and decision was made to commence steroids in the form of methylprednisolone at the dose of 1 mg/kg for 5 days. There were a dramatical clinical improvement with resolution of fever, hemodynamic stability, and decrease in oxygen requirements augmented by a concomitant drop in inflammatory markers leading to discharge from ICU within days and hospital with safe outcomes.

After discharge, the patient was followed up on outpatient basis in cardiology clinic where she remained asymptomatic and clinically well. Her follow‐up evaluation with coronary CT angiography showed no evidence of coronary artery disease. She underwent a cardiac MRI, which showed no evidence of myocarditis with normal left ventricular systolic function with EF of 58%.

## 3 DISCUSSION

Multi‐inflammatory syndrome in children (MIS‐C) was first described in Europe and North America earlier in COVID‐19 pandemic, where multiple children and young adolescents presented with fever and multi‐organ involvement, including the cardiovascular, respiratory, and gastrointestinal system.^6^, ^7^ Although the cause was not clear, the strong association with SARS‐CoV‐2 infection was observed. At the same time, up to 50% of the described cases meet the criteria for complete or incomplete Kawasaki disease.^7^ Since then, multiple criteria were proposed to define MIS‐C.^8^ The exact incidence of MIS‐C is uncertain; however, it appears to be rare. In an early study in New York State, the estimated incidence rate was 0.6% among patient with laboratory confirmed COVID‐19.^6^

Although the majority of cases were reported in children and young adolescents, the syndrome was eventually reported in adults given the similarities in clinical and laboratory presentation highlighted.^5^ For multisystem inflammatory syndrome in adults (MIS‐A), there are set criteria which were suggested by different international health authorities which broadly encompass severe illness in adults >21 year of age that requires hospitalization, recently confirmed COVID‐19 infection within a defined time‐frame, multi‐organ dysfunction with a companied an associated inflammatory response in the absence of infection or alternative active diagnoses (Table 2).^5^, ^9^, ^10^, ^20^ Our patient fulfils the criteria since she presented with recent confirmed evidence of SARS‐CoV‐2 infection with complete clinical recovery within 12 weeks of infection with no evidence of ongoing current COVID‐19 infections could be affirmed by presentation almost 4 weeks from infection with serial negative COVID‐19 PCR tests including deep respiratory samples.

**TABLE 2:** CDC MIS‐A case definition criteria

A patient aged ≥21 years hospitalized for ≥24 h, or with an illness resulting in death, who meets the following clinical and laboratory criteria. The patient should not have a more likely alternative diagnosis for the illness (eg, bacterial sepsis, exacerbation of a chronic medical condition)		
Clinical criteria		Laboratory evidence
Subjective fever or documented fever (≥38.0 C) for ≥24 h prior to hospitalization or within the first THREE days of hospitalization^a^ and at least THREE of the following clinical criteria occurring prior to hospitalization or within the first THREE days of hospitalization^a^. At least ONE must be a primary clinical criterion		The presence of laboratory evidence of inflammation AND SARS‐CoV‐2 infection
A. Primary clinical criteria	B. Secondary clinical criteria	Elevated levels of at least TWO of the following: C‐reactive protein, ferritin, IL‐6, erythrocyte sedimentation rate, procalcitonin A positive SARS‐CoV‐2 test during the current illness by RT‐PCR, serology, or antigen detection
Severe cardiac illness *Includes myocarditis, pericarditis, coronary artery dilatation/aneurysm, or new‐onset right or left ventricular dysfunction (LVEF < 50%), 2nd/3rd degree A‐V block, or ventricular tachycardia. (Note: cardiac arrest alone does not meet this criterion)* Rash AND non‐purulent conjunctivitis	New‐onset neurologic signs and symptoms *Includes encephalopathy in a patient without prior cognitive impairment*, *seizures*, *meningeal signs*, *or peripheral neuropathy (including Guillain*‐*Barré syndrome)*	
	C. Shock or hypotension not attributable to medical therapy (eg, sedation, renal replacement therapy) D. Abdominal pain, vomiting, or diarrhea E. Thrombocytopenia (platelet count <150,000/microliter)	

Abbreviations: CMV, Cytomegalovirus; CRP, C‐Reactive Protein; EBV, Ebstein Barr Virus; EF, ejection fraction; HIV, human immunodefeciency virus; ICU, intensive care unit; LV, left ventricle; MIS‐A, multi inflammatory syndrome in Adult; mRNA, messenger RNA; Pro BNP, pro B‐type natriuretic peptide; RT PCR, Real Time Polemyrase chain reaction; WBC, white Blood cells.

^a^ These criteria must be met by the end of hospital day 3, where the date of hospital admission is hospital day 0.

In order to postulate possible underlying pathophysiological mechanisms, proposed explanations suggested viral antigenic particles provoking intense autoantibodies response or T‐cell directed to host cells, direct immune damage to infected cells displaying viral particles or formation of immune complexes that activate the intense inflammation.^11^

Intriguingly, the patient presented with neck pain and found to have cervical lymphadenopathy during evaluation. Although lymphadenopathy has been highlighted as one of the reported vaccines adverse events, it is usually mild, ipsilateral to injection sites, and not diffuse.^12^ Furthermore, neck pain is very common in MIS‐C and Kawasaki disease reported as high as 25% attributed to retropharyngeal edema and secondary inflammation in the lymphatic system.^13^ In our patient, lymphoreticular inflammation secondary to the underlying immune activation is furtherly supported by the documented hepatosplenomegaly. It would have been more informative if we obtained a tissue sample to ascertain the underlying associative histological pathology.

Of note, in addition to the recent diagnosis of confirmed COVID‐19 disease, the patient received two doses mRNA Moderna vaccine. The first dose predates the infection while the second dose a fortnight afterward when the patient was completely asymptomatic. The need and timing of vaccination schedule in patients following acute SARS‐CoV‐2 remain controversial but most healthcare authorities recommend that following resolution of acute symptoms and discontinuation of isolation.^19^ Moreover, although the diagnosis of the multisystem inflammatory syndrome relating to the highlighted presentation is certainly plausible, it is virtually impossible to ascertain if it is solely attributed to the recent COVID‐19 disease or in relation to the vaccination. The literature describes MIS cases following vaccination secondary to inactivated as well as mRNA vaccines with or without previous COVID‐19 infection coined MIS‐V.^14^, ^15^ Either ways, the role of the immune system in triggering MIS remains vital since antigen presentation and interaction with host immune system is provided through infections or vaccination. SARS‐CoV‐2 S antigen or other related viral antigens seems integral components for provoking the immune system since it the main component utilized by mRNA vaccines such as Modern which has been attributed in other autoimmune pathology.^16^, ^17^

In support of our diagnosis of MIS‐A or V associated either with COVID‐19 infection or vaccination, the patient had repeatedly negative cultures for bacterial pathogens, negative molecular tests for related viral infections together with the absence of any alternative diagnoses including auto immune inflammatory conditions. Furthermore, the dramatic response to the immune suppressive therapy in form of steroids therapy supports an immune based pathology. Those observations although anecdotal do support the role immune suppression as the mainstay therapy for MIS‐C/A/V. While cases are sparse with no strong supporting evidence‐based practice, accumulated evidence supports alternative immune modulating or suppressive therapy that include other forms of steroids, intravenous immune globulins, or interleukin inhibitors such as Tocilizumab or Anakinra.^2^, ^5^, ^18^

## 4 CONCLUSION

Multisystem Inflammatory Syndrome (MIS) is a new clinical entity which has been described with the evolving COVID‐19 pandemic in relation to SARS‐CoV‐2 infections, initially described in the pediatric population (MIS‐C), then adults (MIS‐A), and eventually in relation to vaccinations (MIS‐V). The syndrome is characterized by an intense inflammatory activation that requires hospitalization, involves multisystem presentation with organs dysfunctions in the absence of associative infective or alternative diagnoses. The clinical condition has significant morbidity but relatively lower mortality. Early recognition and institution of immune suppressive therapy seems the mainstay for safe outcomes.

## CONFLICT OF INTEREST

The authors declare no conflicts of interest.

## AUTHOR CONTRIBUTIONS

Ahmad Al Bishawi involved in conceptualization, writing—original draft, and data curation. Maisa Ali involved in data collection, data analysis, manuscript writing, and supervision. Khaled Al‐Zubaidi involved in data collection, data analysis, and manuscript writing. Hamad Abdelhadi involved in conceptualization, writing—review and editing, and supervision.

## ETHICAL APPROVAL

Consent form was obtained from the patient toward academic publication. The case report received approval from HMC medical research center (MRC) for publication under MRC‐04‐21‐516.

## CONSENT

Written informed consent was obtained from the patient to publish this report in accordance with the journal's patient consent policy.

## Data Availability

The data that support the findings of this study are available on request from the corresponding author. The data are not publicly available due to privacy or ethical restrictions.
